# Group Telehealth Interventions Fostering Social Connection Among Older Veterans and Their Families

**DOI:** 10.1093/geroni/igab046.789

**Published:** 2021-12-17

**Authors:** Rachel Weiskittle, Michelle Mlinac

**Affiliations:** 1 University of Colorado Colorado Springs, Colorado Springs, Colorado, United States; 2 VA Boston Healthcare System, Boston, Massachusetts, United States

## Abstract

During the early months of the COVID-19 pandemic, virtual and telephone visits rapidly replaced most in-person care within the Veterans Health Administration (VA) to reduce virus spread. To address the emerging mental health needs of older Veterans (e.g., isolation, loneliness), we developed an 8-week group treatment manual, deliverable over telephone or videoconference, to foster social connection and address pandemic anxieties. The manual was disseminated in March 2020 as a rapid response to emergent COVID-19 pandemic realities, during which many locations in the United States called for immediate self-quarantine measures for unknown durations. This talk will present the user-centered design of the manual, preliminary feasibility and acceptability findings from provider surveys, and introduce versions of the manual targeting specific populations (e.g., caregivers, Spanish speakers) currently in development or in pilot testing.

